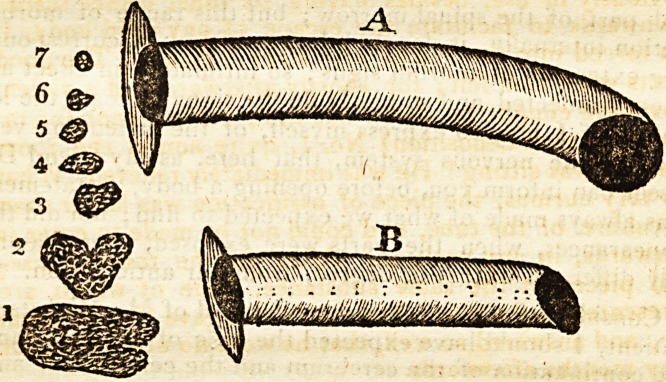# Case of Cynanche Laryngea, Requiring Tracheotomy, and the Continued Use of a Canula Ever Since the Operation

**Published:** 1818-01

**Authors:** 


### Medico-Chirurgical Journal and Review.

VOL. V.] JANUARY, 1818. [NO. 25.

### PART I.

### ORIGINAL COMMUNICATIONS.

——— quid utile ———

### *Case of Cynanche Laryngea, requiring Tracheotomy, and the continued Use of a Canula ever since the Operation.*

( With a Drawing. )

MR. PRICE, jeweller, residing in King's Terrace, Southsea, near Portsmouth, about 30 years of age, and previously unaffected with any complaint of the throat or chest, excepting occasional palpitation of the heart, and sense of uneasiness at the epigastrium, had been for a few days, in the beginning of October 1816, affected with *Cynanche Tonsillaris,* which was removed by the common antiphlogistic measures. But after exposure to the night air, the inflammation suddenly returned, and now fell on the glottis and other points of the larynx, causing a dreadful sense of suffocation. The pulse was, at this time, little altered, excepting from the alarm into which the patient was thrown. On examination, the epiglottis was brought into view, and, with the neighbouring parts, appeared inflamed. Leeches were immediately applied round the throat, and he was bled from the arm, *ad deliquium.* Blisters succeeded the leeches; and the most powerful cathartics were exhibited. The vapour of warm water and vinegar was inhaled; and the patient by these means was soon relieved. In three days a copious discharge of *apparently*

*purulent matter* took place from the throat, brought off by hawking and coughing, and giving rise to apprehensions that the previous inflammation had terminated in suppuration or ulceration. This discharge amounted, at one time, to a tea-cup full in twenty-four hours. His breathing was pretty free; but still he had an unpleasant *vox rauca:* he looked ill, and had some pyrexia. The discharge, however, gradually lessened; his appetite returned; and it became evident, that what appeared to be purulent matter, was only a secretion from the inflamed parts.

When things were in this favourable train, an accidental exposure to a stream of cold air, was almost immediately succeeded by such a difficulty of respiration, as baffled every energetic measure that could be devised for its removal.

*November 5th,* 1816. In consultation, Dr. Lara, Dr. Denmark, and Dr. Johnson. The respiration is most difficult; eyes prominent; cheeks flushed; lips livid; forehead and breast covered with a cold, clammy perspiration; countenance ghastly, and expressive of unutterable and indescribable sufferings; both sides of the larynx, to below the cricoid cartilage, tender and swelled; the whole neck tumefied, and red from the sinapisms that had been applied. Death was now rapidly approaching; and it was very doubtful whether effusion in the lungs had not already taken place. It was unanimously agreed that nothing but tracheotomy could save, or, even for a short time, prolong life. It was also feared by two of the three medical attendants, that such organic derangement had already supervened in the larynx, as would render the operation unsuccessful. But this was no solid reason for abstaining from the *anceps remedium.*

*Operation,* 5, P. M. Dr. Denmark took the knife. An incision through the integuments, from the lower edge of the thyroid gland to within half an inch of the sternum, was made, as nearly as could be guessed, on the line that divides the sterno-hyoid and thyroid muscles of the opposite sides. When these muscles were exposed, however, no such line of division could be perceived, owing, no doubt, to the tumefaction and inflammation of the parts. The incision was therefore carried cautiously through these muscles in a direction for the trachea. The motion of these muscles, and of those of the neck in general, but of deglutition in particular, was violent, and extremely embarrassing. Several venous and arterial branches were cut, and bled freely, but ceased to bleed without ligature.

Anxious to come at the trachea as near the thyroid gland as possible, the right lobe of that body was accidentally entered, in consequence of the convulsive action of the muscles. A principal branch of the inferior thyroid artery of that side sprang, and bled profusely. Dr. Denmark plunged a tenaculum as nearly as possible on it, and Dr. Johnson affixed a ligature. The hæmorrhage continued; the ligature was instantly cut away; another plunge was made with the tenaculum, and now the ligature proved successful. It was not thought advisable to advance farther in that direction, and the enlargement of the wound downwards towards the sternum required caution, lest, in the struggles for breath, some large vessel in that vascular neighbourhood should be divided. At length, after a somewhat tedious dissection, the difficulty of which can only be appreciated by those who see it performed on a living subject gasping and struggling for breath, the trachea was laid bare, at full an inch from the surface. Those who talk about cutting out a piece of a tracheal ring at the bottom of such a cavity as this, while the trachea itself is in constant motion, and the surrounding muscles ever altering their relative positions in the efforts at respiration, have probably never practised it. The tube was slit open, with the point of a double edged scalpel, about a third of an inch, when the hissing of the air, backwards and forwards, became very loud. With some difficulty a flattened silver canula was introduced, when the relief, instantaneously experienced by the unhappy sufferer, may be conceived, but cannot be described. He could hardly be freed from his bloody garments, and propped up in bed, when he fell into a profound sleep! The canula was secured by means of tapes, adhesive straps, &c. and he slept and breathed tranquilly, till nine o'clock at night, when a fit of coughing displaced the tube, and threw him into great distress as well as danger. It was found that the longitudinal opening in the trachea admitted of but a very inadequate ingress and egress of air, when the tube was out; and the introduction of the latter at the bottom of a deep sinus, as the wound was, presented some difficulty. This difficulty was a good deal overcome by the suggestion of Dr. Johnson, viz. that of pushing the end of an elastic gum catheter a little way through the silver canula, so as to make a *blind end*, which readily went into the opening of the trachea, when the gum catheter was immediately withdrawn, and the canula left in. A sound and refreshing night's sleep succeeded this long period of restlessness and misery.

*November 6th.* This morning the irritation of a foreign body had caused a great secretion of phlegm and mucus throughout the larynx and trachea, and the efforts to cough it up by the mouth and through the canula were very distressing, and required constant attention. The canula was repeatedly displaced. Voice and speech were entirely gone of course; but deglutition was easy, and he this day took some brisk purgative medicine. The countenance had regained much of its pristine serenity ever since the operation; the pulse was regular, but quick; and the symptomatic fever was not considerable. His spirits are now good, and he is constanstly writing down and answering questions. By the evening of this day, considerable tumefaction had taken place round the wound; the cavity was consequently rendered deeper; the canula, become too short, was every minute displaced, and would no longer answer. It was always necessary when the tube was removed, to draw one edge of the slit in the trachea apart from the other, by means of a crooked probe or hook, when the patient could breathe with tolerable ease. But the mucous secretion was most tiresome and embarrassing; so much so, that frequently the convulsive struggles to force it up appeared almost decisive of his fate! During the night, a new tube, adapted to the depth of the wound, was many times thrown out, and a surgeon was obliged to be in constant attendance. A strong mercurial fœtor this day was discovered on the breath, from some calomel which he had taken with purgative medicine, during the previous inflammation.

*7th.* This day a very smart ptyalism developed itself, and the increased flow of saliva from the mouth and fauces added greatly to the patient's other sufferings. The inflammation, however, and tumefaction around the wound were beginning to subside; and, for the first time since the operation, he could articulate a little. When the tube was now displaced by accident or design, the dyspnœa was not quite so distressing as at first; and upon the whole, a gleam of hope broke through the clouds, and gave anticipation of final success. The bowels were kept very free to day, in hopes of checking the ptyalism, which was still profuse; but the countenance was cheerful and serene, excepting during a paroxysm of coughing.

*8th.* Has passed a good night; the tube was twice displaced by coughing, but easily reinstated. This morning, the artificial passage was closed with the finger, and he breathed by the mouth; but the difficulty was as great as previous to the operation; so that nothing but time has yet been gained. The canula of a common trochar introduced on a bougie that just fills its calibre, answers best now.

*9th.* Has had a good deal of fever last night; and complained of pain and coldness of the lower extremities. Expectorates much viscid phlegm and mucus, which have a very disagreeable fœtor.

*10th.* A feverish and restless night; was sometimes delirious; but on taking some active purgatives, he has had four copious stools, and the febrile symptoms have subsided.

*11th.* Passed a good night; has no fever; breathes comfortably through the tube and mouth together; and could blow out a candle by the air from his mouth alone, of which he was highly proud. Can speak pretty plain. Inhaled warm aqueous vapour through the tube, which he found very pleasant.

*12th, 13th, 14th.* Little or no alteration.

*15th.* Took out the tube to-day, and closed the wound completely with the finger. The difficulty of breathing was, at first, great; but in a little time it became somewhat easier; and he breathed nearly ten minutes through the natural passage. He is becoming very hoarse, but he sleeps quietly the whole night, and only changes the tube twice in twenty-four hours.

*20th.* He now eats, drinks, and sleeps well. The tube is every day taken out, and he is made to breathe through the natural passage, as long as he conveniently can; but it is evident that the original obstruction remains. He expectorates some semi-purulent looking mucus, which smells very badly.

*29th.* The tube has only been changed every second day since last Report. On withdrawing it to day, he breathed with great difficulty, and we were forced to introduce it with considerable dispatch, as he seemed to be suffocating. There is now every appearance of a tedious or doubtful issue, after all our exertions!

*December 16th.* Six weeks have now elapsed since the operation. His voice is scarcely audible. He cannot breathe at all, but through the tube. We tried to pass down a curved elastic tube through the rima glottidis, but without success. A finger was passed behind the epiglottis, but the opening into the larynx could not be felt; neither could any morbid structure or ulceration be discovered. Linimentum hydrargyri was directed to be rubbed over the region of the larynx, which evidently brought on a slight ptyalism. An occasional intermission of the pulse is now observable, and he has palpitation of the heart.

*30th.* Or fifty-six days after the operation, he coughed up through the tube a piece of apparently carious bone [No. 3.] of a cancellated structure, and supposed to be a piece of what is termed ossified thyroid or cricoid cartilage. In other respects he is much the same as before.

*January* 1*st*, 1817. Last night he felt a piece of bone fall down into the lungs, and has ever since been in a dreadful state of coughing. He feels the piece come up to the tube, but as it cannot get out, it falls back, and keeps him in constant agitation. Something decisive must now be done. The Canula A was therefore quickly constructed, and being much larger in the bore than the one previously in use, besides having its inner orifice turned downwards to facilitate the escape of any solid body, it was forced into the wound with some difficulty. The good effects were soon felt; for shortly afterwards, in a violent convulsive cough, he threw out the piece of bone [or rather calcareous deposition] No. 1, quite across the room where he was sitting! He was immediately relieved. What was very curious, the piece of ossification was larger than the calibre of the tube, and could not be made to enter it afterwards, in any position. After this he threw out several pieces of the same substance, some of which are represented in the plan: but the tube A gave him so much ease, and answered so well, that he wore no other kind for many months afterwards.

*March* 18*th*, 1817. Has continued in good health since last Report, and can breathe a few minutes with the tube corked, which is the usual way of exercising the natural passage. He went to London this day, and consulted Mr. Astley Cooper and others, who recommended patience, fumigations, &c. but did not think that any thing in the operation way could be done.

He remained a month or six weeks in London; and then returned to this place. By corking the tube several times a-day, he at last acquired the power of keeping it so, and of breathing through the mouth for several hours at a time; but he thought it hurt his health; and, latterly, he seldom stops the tube, excepting when he wishes to speak, which he can do plainly enough by placing his finger on the tube. The canula B. is that which he has worn for the last two or three months, and it gives little or no uneasiness.

*December 5th,* 1817. ONE YEAR AND ONE MONTH have now elapsed since the operation was performed; and the tube is worn without inconvenience, being merely taken out twice a week to clean, and immediately replaced. His health is as good as at almost any period of his life. There is a slight tumefaction, and also a considerable induration around the wound, from which a discharge proceeds as if from an issue. The actual site or nature of the obstruction in the larynx cannot be ascertained; and nothing but time can determine the result of this case, which stands unique in the annals of surgery."

## Figures and Tables

**Figure f1:**